# Three Dimensional Approaches to Personality Disorders: a Review on Personality Functioning, Personality Structure, and Personality Organization

**DOI:** 10.1007/s11920-021-01250-y

**Published:** 2021-06-28

**Authors:** Susanne Hörz-Sagstetter, Ludwig Ohse, Leonie Kampe

**Affiliations:** 1 Psychologische Hochschule Berlin (PHB), Am Köllnischen Park 2, 10179 Berlin, Germany; 2 Zentrum für Psychosoziale Medizin, Klinikum Itzehoe, Germany

**Keywords:** Personality disorders, Personality functioning, Personality structure, Personality organization, Level of Personality Functioning Scale, Review

## Abstract

**Purpose of Review:**

The concept of personality functioning (Alternative DSM-5 Model of Personality Disorders) has led to increased interest in dimensional personality disorder diagnosis. While differing markedly from the current categorical classification, it is closely related to the psychodynamic concepts of personality structure and personality organization. In this review, the three dimensional approaches, their underlying models, and common instruments are introduced, and empirical studies on similarities and differences between the concepts and the categorical classification are summarized. Additionally, a case example illustrates the clinical application.

**Recent Findings:**

Numerous studies demonstrate the broad empirical basis, validated assessment instruments and clinical usefulness of the dimensional concepts. Their advantages compared to the categorical approach, but also the respective differences, have been demonstrated empirically, in line with clinical observations.

**Summary:**

Evidence supports the three dimensional concepts, which share conceptual overlap, but also entail unique aspects of personality pathology, respectively.

## Introduction

The contemporary classification of mental disorders—DSM-5 [1] and ICD-10 [2]—is based on the assumption that personality disorders (PDs) are a set of *categorical* and qualitatively distinct entities. Since its introduction 40 years ago [3], this conceptualization has been challenged by numerous studies suggesting that PDs are not categorical but *dimensional* constructs [4–6], i.e., they can be located on a continuum from well-functioning to highly dysfunctional personality. In addition, the current classification has serious limitations, such as high comorbidity between, and high heterogeneity within PDs [7–9], indicating that the notion of PDs as separate constructs may not be valid. The categorical system is considered to hinder progress in research and deemed insufficient for use in clinical practice [9–11].

In an effort to address these shortcomings, the DSM-5 Work Group for Personality and PDs proposed the Alternative DSM-5 Model for PDs (AMPD) [1, 12]. The AMPD was meant to replace the categorical DSM classification, but was ultimately not adopted to the current official diagnostic codes [13], while the previous criteria of DSM-IV [14] were maintained for DSM-5. The essential requirement for the presence of a PD are impairments in *personality functioning* (Criterion A) [15], accompanied by a manifestation of *pathological personality traits* (Criterion B) [16]. The AMPD has stimulated plenty of research [17••] and has led to increased interest in dimensional assessments of PDs in general. This includes other new models, such as the PD classification proposal for ICD-11 [18], and previously existing approaches, such as the concepts of *personality structure* [19] and *personality organization* [20].

Personality structure and personality organization are psychoanalytic concepts that substantially influenced the development of the concept of personality functioning [15]. The three concepts differ fundamentally from the current categorical classification of PDs in that they define core pathology dimensions based on disturbances in the self and interpersonal relations, which can be rendered to one global continuum, spanning levels from normal to severely disturbed functioning of personality. While differences between the categorical classification of PDs and dimensional approaches have been reviewed before on a theoretical level [7, 21], no review has yet compiled empirical studies on that matter (focusing on personality functioning, personality structure, and personality organization). It is furthermore noteworthy, that—despite common ground—personality functioning has also notable conceptual differences to personality structure and personality organization, which have been pointed out in previous reviews from the perspective of Operationalized Psychodynamic Diagnostics (OPD) [22••] and object relations theory [23•]. However, these reviews were, again, theoretical and whether these concepts are also *empirically* distinguishable has not been subject of a review before.

Addressing the aforementioned issues, the current paper aims to review studies on personality functioning, personality structure, and personality organization with a focus on categorical PDs and similarities and differences among the concepts themselves. For this purpose, the concepts are briefly introduced along with their empirical background, assessment instruments, and their clinical relevance, each followed by a review of the studies linking the respective concept with PDs. Studies that investigated associations and differences between the three dimensional concepts themselves are subsequently summarized. Finally, a case example of a patient assessed with interview measures for personality functioning, personality organization, and the categorical classification of PDs is presented to illustrate the advantages and disadvantages of the different approaches in clinical practice.

## Dimensional Approaches to Personality Disorders: Personality Functioning, Personality Structure, and Personality Organization

### Personality Functioning

#### Conceptual Framework

Personality functioning is operationalized by the Levels of Personality Functioning Scale (LPFS), which was constructed by synthesizing previously existing measures (with different theoretical underpinnings) of general personality psychopathology [15]. The LPFS consists of four dimensions: *identity*, *self-direction*, *empathy*, and *intimacy*; the former two reflecting *self functioning*, the latter two reflecting *interpersonal functioning*. These dimensions span five levels of impairment (0 = “no,” 1 = “some,” 2 = “moderate,” 3 = “severe,” and 4 = “extreme”) and are rendered to a single continuum that aims to cover the core pathology of all PDs. According to the AMPD, a moderate or greater impairment (level of 2 or larger) in personality functioning is the essential criterion (Criterion A) for diagnosing a PD. The second major component of the AMPD, pathological personality traits (Criterion B), captures disorder-specific characteristics beyond general personality psychopathology [16]. Though the AMPD pursues primarily a dimensional approach, it enables the classification of six specific PDs (antisocial, avoidant, borderline, narcissistic, obsessive-compulsive, and schizotypal), which are characterized by specific impairments in personality functioning.

#### Empirical Background

Zimmermann et al. [17••] reviewed a number of studies on reliability and structure of personality functioning, such as on convergent validity with a variety of constructs, including other PD conceptualizations, (pathological) personality traits, various indicators of mental and physical dysfunction, and interpersonal problems. Further reviews discussed personality functioning against the background of other scientific frameworks, such as the categorical DSM classification [7, 21], psychodynamic models [24], object relations [23•], interpersonal theory [25], schema therapy [26], stress [27], neurobiology [28], and taxonomy of psychopathology [29].

#### Instruments

The LPFS itself serves as an expert rating instrument. Prominent self-report measures are the LPFS–Self Report [30], the LPFS–Brief Report [31, 32], the DSM-5 Levels of Personality Functioning Questionnaire [33], the Levels of Personality Functioning Questionnaire for Adolescents from 12 to 18 years [34], and the Self and Interpersonal Functioning Scale [35]. Structured interviews have only lately been developed, including the Clinical Assessment of the LPFS [36], the Semi-Structured Interview for Personality Functioning DSM-5 (STiP-5.1) [37], and the Structured Clinical Interview for the DSM-5 Alternative Model for Personality Disorders–Module I (SCID-5-AMPD-I) [38]. Recent reviews compiled evidence for the validity and reliability of these measures [17, 39].

Regarding the interviews for personality functioning, most evidence is emerging for STiP-5.1 [37, 40] and SCID-5-AMPD-I [41•, 42, 43•, 44]. Both interviews split each of the four LPFS domains into three subdomains (resulting in 12 subdomains in total). For example, in the SCID-5-AMPD-I, the domain identity is divided into “sense of self,” “self-esteem,” and “emotional range and regulation”. Each subdomain is rated on a scale from 0 (“no impairment”) to 4 (“extreme impairment”). The ratings of the main domains are obtained by averaging the ratings of the respective subdomains, while the overall rating of personality functioning results by averaging all 12 subdomains.

#### Clinical Relevance

Personality functioning was explicitly designed to enhance clinical utility compared to the current categorical classification of PDs [12]. Correspondingly, a recent review found evidence that the AMPD as a whole, including personality functioning (Criterion A) and pathological personality traits (Criterion B), provides extensive information for case conceptualization and clinical decision-making [45]. Hopwood [46] elaborated a comprehensive framework for using the AMPD in clinical practice. It is important to note that the LPFS seems to be relatively easy to learn and apply, as inexperienced students were able to utilize the LPFS in an acceptable manner [47•, 48–51]. The concept of personality functioning substantially influenced the upcoming ICD-11 classification and its severity rating of personality pathology [18, 52], which emphasizes its great importance for the current paradigm shift in PD diagnosis.

#### Personality Functioning and Personality Disorders

Personality functioning and categorical DSM PDs have been investigated in numerous studies [31, 32, 34, 35, 37, 40, 42, 43•, 47•, 48, 51, 53–67]. Several tested their convergent validity and, overall, confirmed it [31, 32, 37, 43•, 51, 55, 57, 59–65, 67]. While most studies examined general impairments in personality functioning, some also explored disorder-specific impairments, and found convergence with DSM-IV for antisocial [56], avoidant [61, 65], and obsessive-compulsive [57, 61] PD, such as for all six AMPD PDs in a further study [64]. These findings suggest that personality functioning encompasses a broad range of personality psychopathology, but also indicate that the AMPD may be able to replace the DSM-IV PD classification and at the same time keep a reference framework for it. An important contribution in this regard is an interview-based study of 282 patients, which came to the conclusion that a global LPFS score of 1.5 may be a more reasonable threshold for detecting a DSM-IV PD compared to the threshold of 2 (“moderate impairment”) given in Criterion A of the AMPD [43•].

In the light of the foregoing, it is important to stress that personality functioning is not meant to be congruent with DSM-IV PDs, but to overcome the shortcomings of their categorical nature [7]. Addressing this issue, two studies found that personality functioning was a better predictor for psychosocial functioning than DSM-IV PDs [42, 68]. In another study, impairments specific to antisocial PD added incremental validity over the corresponding DSM-IV category in predicting psychopathy [56]. Elsewhere, personality functioning (especially self functioning) was a better predictor for dropout in psychotherapy for PDs than the DSM-IV categories [58]. These findings illustrate that the concept of personality functioning is beginning to empirically prove its expected added value over the current categorical classification of PDs. However, evidence in this matter is far from being sufficient and future studies should examine possible advantages of personality functioning over DSM-IV PDs (and vice versa) on a variety of criterion variables.

## Personality Structure

### Conceptual Framework

The concept of personality structure is rooted in psychodynamic/psychoanalytic theory, going back to Freud’s structural model [69, 70] and developed further by many other theories [71–73]. According to a modern definition, personality structure refers to the “availability of mental functions for the regulation of the self and its relationships to internal and external objects” (p. 199) [19]. The construct of personality structure cuts across the spectrum of PDs and also complements the descriptive classification of other mental disorders.

A frequently used framework for the assessment of personality structure is the Operationalized Psychodynamic Diagnostics (OPD) [19]. The OPD is an interview-based multi-axial system of psychodynamic diagnosis spanning four axes: axis I = “experience of illness and prerequisites for treatment,” II = “interpersonal relations,” III = “conflict,” IV = “structure,” and V = “mental and psychosomatic disorders.” The fourth axis, also referred to as OPD levels of structural integration axis (OPD-LSIA), was developed by integrating several psychodynamic concepts into a functional description of personality structure [74] and has the goal to capture it using clinical observations and remaining close to the individual’s behavior in relation to the interviewer. OPD-LSIA is composed of ratings of the following eight domains: “self-perception,” “object perception,” “self-regulation,” “regulation of the object relationship,” “internal communication,” “communication with the external world,” “attachment to internal objects,” and “attachment to external objects.” Each domain is rated on a four-point scale, ranging from “high level of structural integration,” to “moderate,” “low,” and “disintegrated level of integration,” also using intermediate levels. Based on the domain ratings, a global level of structural integration is assigned.

### Empirical Background

Recent studies on the commonly used OPD-LSIA yield acceptable internal consistency, good inter-rater reliability and validity [22, 75••, 76]. Convergent validity of the OPD-LSIA was confirmed in studies comparing it to other self-report and observer informed instruments focusing on related constructs like the Reflective Functioning Scale [77] or the psychodiagnostic chart of the Psychodynamic Diagnostic Manual (PDM) [78]. The approach of the OPD-LSIA, using a dimensional profile of individual structural impairments, also shows added value in the diagnosis of other mental disorders, e.g., eating disorders [79], chronic pelvic pain syndrome [80], or anxiety [81].

### Instruments

Besides the OPD-LSIA [19], several interview-based methods to assess personality structure have been developed. Among others, these are the P-axis of the PDM [82], the Karolinska Psychodynamic Profile [83], and the Scales of Psychological Capacities [84].

Personality structure according to the OPD-system can be assessed by interview format or as self-report with the OPD Structure Questionnaire (OPD-SQ) [85, 86]. OPD-LSIA and OPD-SQ show good convergence and both are good predictors for DSM-IV PDs [87]. Recently, a 12-item short version of the questionnaire was developed [88]. The OPD-SQ is closely related to other self-reports for psychodynamic conceptualizations of personality pathology [89].

### Clinical Relevance

Achieving changes in personality structure is one of the primary goals of psychodynamic psychotherapy, particularly for patients with PDs. Henkel et al. [90] demonstrated the clinical usefulness of the OPD-LSIA complementing the classificatory PD diagnoses. Based on the concept of personality structure and closely related to the OPD-LSIA, a therapeutic approach was developed by Rudolf [91]. The OPD-LSIA can be used to identify the most significant structural difficulties, define therapy goals, and plan treatment strategies [92]. In order to assess structural change beyond symptoms, the Heidelberg Structural Change Scale (HSCS) can be employed [93–95]. Using the OPD axes, problem foci are chosen and the patient’s level of awareness for these problem areas across time is scored using the HSCS. This method makes it possible to quantify structural change on the scale. The OPD-LSIA and the OPD-SQ have been employed in cross-sectional and longitudinal studies [93, 95–97].

### Personality Structure and Personality Disorders

PDs can be located at different levels of personality structure. As the OPD-LSIA captures main features of personality pathology, higher levels of severity in PDs are associated with lower levels of personality structure. For example, a study comparing OPD-LSIA and diagnoses from the Structured Clinical Interview for DSM-IV–Axis II PDs (SCID-II) [98] found that cluster B patients (histrionic, narcissistic, borderline, and antisocial PD) had a significantly lower level of personality structure than cluster C patients (avoidant, dependent, and obsessive-compulsive PD). OPD-LSIA ratings of patients with PDs indicated significantly more impairment than of patients without PDs [75••]. A meta analysis [22••] of eight studies on the interrelation between severity of personality pathology according to the OPD-LSIA and categorical DSM PDs reported a moderate to large effect size of *r* = 0.42 (with a 95% confidence interval of 0.32 to 0.51). Recent studies testing the relationship between personality pathology according to the OPD-LSIA and severity of PD diagnoses showed a strong correlation [47•, 75••, 86]. Severity of personality pathology according to the OPD-SQ was higher for patients with comorbid major depression and borderline PD than for patients with major depression only [99], and accounted for differences in negative emotions between these two patient groups [100].

## Personality Organization

### Conceptual Framework

The concept of personality organization is based on the object relations theory framework by Kernberg [73, 101, 102]. In his fundamental theoretical works, Kernberg derives distinct domains and levels of impairment in psychological functioning from etiologic considerations about the development of the personality and draws conclusions on personality pathology. Levels of personality organization are assessed across the domains identity, primitive defense mechanisms, and reality testing (later the dimensions object relations, aggression, rigidity and coping, and moral values were added), and span across a continuum according to severity, ranging from normal/neurotic personality organization (NPO) to borderline personality organization (BPO) to psychotic personality organization (PPO). While normal/NPO includes an integrated identity, use of mostly mature defense mechanisms and stable reality testing, BPO is characterized by impaired identity, the use of mostly primitive defense mechanisms, and fluctuating difficulties with reality testing. For PPO, the suspension of reality testing is a core feature. Although some PDs can also be found at the level of NPO, the threshold for fulfilling a severe PD is the level of BPO. Its foundation in psychoanalytic theory framework is an outstanding feature of the personality organization model that essentially shapes its operationalization [103, 104].

### Empirical Background

The three core domains, “identity,” “primitive defenses,” and “reality testing,” have been found to be internally consistent [105••]. Furthermore, several studies demonstrated convergent validity of the individual domains [106–109]. On the basis of diagnostic results, a prototypical profile for BPO was developed and its ability to discriminate between BPO and non-BPO tested successfully [110, 111]. The distinct levels of personality organization have successfully proven to display distinct content of impairment [112–114] and to differentiate between neurotic, borderline, and schizophrenic patients [115]. The levels relate to psychiatric severity of pathology [41•, 113, 116•], psychological distress, severity of symptoms [117–120], mental health, psychosocial functioning [121•], and reflective functioning [122].

### Instruments

Several instruments have been developed to assess the levels of personality organization across the domains [123]. As self-report measure, the Inventory of Personality Organization (IPO) [124, 125] captures important elements of the concept. Different short versions of the IPO have been developed, varying in item numbers and underlying factor structures [126–134]. Another self-report measure of personality organization is the Borderline Personality Inventory [135].

For a thorough and complete assessment of personality organization, Kernberg et al. [115] underline the importance of the clinical impression and the advantages of interview-based methods compared to self-report methods. Based on the initial clinical interview, the Structural Interview [136], the Structured Interview of Personality Organization (STIPO) [137], was developed for the assessment of six levels of personality organization (normal, neurotic I, II, borderline I, II, III) across the domains identity, object relations, primitive defenses, coping and rigidity, aggression, moral values, and reality testing [138]. The STIPO shows overall good psychometric properties [105••, 139, 140]. To further meet the needs of clinical reality, a short version [141] has recently been introduced and is currently under empirical investigation.

Other approaches to the assessment of personality organization use narrative analyses, based on interview or projective techniques, to assess the developmental level of object relations and their mental representations. Examples are the Social Cognition and Object Relations Scale [142–145], the Concept of the Object on the Rorschach Scale [146, 147], and the Quality of Object Relations Scale [148]. Further scoring systems are the Personality Organization Diagnostic Form [149, 150] and a theory-driven profile interpretation of the Dutch Short Form of the Minnesota Multiphasic Personality Inventory [151–153].

### Clinical Relevance

Studies have shown that the domains and levels of personality organization are useful for the assessment of the patient’s commitment to therapy and willingness/ability to change [138, 154–157, 158••, 159]. Overall, higher levels of personality organization (less severe impairment) were associated with better treatment outcome [160], whereas patients with PPO and low levels of BPO (more severe impairment) were more likely to drop out of treatment [161], associated with helpless and overwhelmed emotional responses by the therapists [162], as well as difficult countertransferential reactions, reduced quality of the therapeutic alliance [163], and non-completion to treatment [160, 164]. Short-term psychotherapy has proven to be more effective for NPO patients, whereas long-term psychotherapy was more effective at long-term follow-up for patients with low personality organization [165]. Also, changes in personality organization have been shown to increase over the course of treatment [166]. Changes in personality organization predicted both symptom and personality improvement during psychotherapy [167, 168].

Based on the theoretical framework of personality organization, Transference-focused Psychotherapy (TFP) [169, 170] was developed for the treatment of severe PDs. By using the techniques clarification, confrontation, and interpretation of transference manifestations, this manualized approach aims to systematically change the underlying personality impairment. TFP has been intensely studied and overall shown efficacy with regard to changes in mentalization, attachment styles, and structural impairments of the personality [155, 171–181].

### Personality Organization and Personality Disorders

The localization of severe PDs at BPO level has been evidenced in multiple studies: compared to patients with no PD, patients with PD revealed significantly lower levels (more severe impairment) of personality organization [105•, 118, 134, 139, 140, 182]. Low levels of personality organization were found found to be related with distinct features crucial for severe PDs, such as self-harm, interpersonal problems, symptomatic distress, severity of depression, and a declined ability to give differentiated and related descriptions of the self and significant others [116•]. Furthermore, the differentiation of the levels of personality organization between normal population, patients with affective and behavioral disorders, and personality-disordered patients was shown in many studies [122, 126, 156, 160, 171, 183–185].

Various studies found differences between DSM PD clusters and distinct levels and domains of personality organization: cluster A (schizoid, schizotypal, paranoid PD) and cluster B (borderline, narcissistic, histrionic, antisocial PD) patients yielded significantly lower levels of personality organization than cluster C patients (avoidant, dependent, obsessive-compulsive PD); differences remained significant when controlling for general severity of psychopathology [140]. While all PDs were associated with a reduced quality of object relations, cluster A PDs mainly correlated with the use of primitive defenses and reality testing distortions, cluster B PDs additionally with lack of moral values, and cluster C PDs with maladaptive coping and character rigidity, identity, and reality testing impairment [186••]. When controlled for clinical distress, correlations between personality organization and cluster C PDs showed to be merely state-dependent, whereas correlations between personality organization and cluster A and B PDs remained strong [187].

Concerning the content of the domains, studies suggest that especially the domains identity, aggression, primitive defenses, and reality impairment depict specific areas crucial for PDs: among all domains of personality organization, these scales showed the highest associations with the number of borderline PD criteria [186••], and correlated highly significant with external measures of clinical severity, even when controlled for overall personality functioning [41••]. For example, the use of primitive defenses was shown to be a crucial domain in PDs, especially with antisocial features [109, 188–192]. These scales are solely operationalized by the concept of personality organization, originating from its theoretical foundation.

## Personality Functioning, Personality Structure, and Personality Organization

A recent factor analytic study suggests that personality functioning, personality structure, and personality organization share a strong common factor [193]. Accordingly, several studies found high convergence between personality functioning and personality organization [35, 40, 41•, 48, 58, 194–196], personality functioning and personality structure [22, 40, 47•, 197], and personality structure and personality organization [40, 78, 89, 140, 198–200]. It is noteworthy that studies successfully used recorded interviews for personality organization [48, 59] and personality structure [47•] to assess personality functioning, suggesting that these concepts have sufficient informational overlap to extrapolate from one to the other (cf. Table 1).

**Table 1:** Comparison of categorical and dimensional concepts of personality psychopathology

	Framework of reference	First occurrence	Approach of classification	Degree of theoretical reference	Examples of operationalization	
					Interviews	Questionnaires
Categorical personality disorders	Official diagnostic codes of DSM-III to DSM-5	1980 [14]	Categorical with 10 entities	a-theoretical	SCID-5-PD	SCID-5-SPQ
Personality functioning	Alternative DSM-5 model for personality disorders	2011 [15]	Dimensional with five levels	Theory-informed	CALF, SCID-5-AMPD-I, STiP-5.1	LPFS-BF, LPFS-SR, DLOPFQ, LoPF-Q 12–18, SIFS
Personality structure	Operationalized Psychodynamic Diagnostics	1996 [201]	Dimensional with four levels	Theory-framed	OPD-LSIA	OPD-SQ
Personality organization	Object relations theory	1967 [102]	Dimensional with three levels	Theory-based	SI, STIPO	IPO, BPI

*DSM* Diagnostic and Statistical Manual of Mental Disorders, *BPI* Borderline Personality Inventory, *CALF* Clinical Assessment of the Level of Personality Functioning Scale, *DLOPFQ* DSM-5 Levels of Personality Functioning Questionnaire, *IPO* Inventory of Personality Organization, *LoPF-Q 12–18* Levels of Personality Functioning Questionnaire for Adolescents from 12 to 18 Years, *LPFS-BF* Level of Personality Functioning Scale–Brief Form, *LPFS-SF* Level of Personality Functioning Scale–Self Report, *OPD-LSIA* Operationalized Psychodynamic Diagnostics–Levels of Structural Integration Axis, *OPD-SQ* Operationalized Psychodynamic Diagnostics–Structure Questionnaire, *SCID-5-PD* Structured Clinical Interview for DSM-5–Personality Disorders, *SCID-5-SPQ* Structured Clinical Interview for DSM-5–Screening Personality Questionnaire, *SCID-5-AMPD-I* Structured Clinical Interview for the Level of Personality Functioning Scale–Module I, *SI* Structural Interview, *SIFS* Self and Interpersonal Functioning Scale, *STiP-5.1* Semi-Structured Interview for Personality Functioning DSM-5, *STIPO* Structured Interview of Personality Organization

Up to the present day, one study used construct-specific interviews for both, personality functioning and personality organization [41••]. That study delivers preliminary evidence of differential properties of these constructs in relation to clinical criteria, as “identity,” “primitive defenses,” “aggression,” and “reality testing” (subdomains of personality organization) were significantly correlated with the number of suicide attempts when controlling for overall personality functioning. Conversely, “empathy” (subdomain of personality functioning) was significantly correlated with the severity of diagnosis when controlling for overall personality organization. Concerning discriminant features of personality functioning and personality structure, an expert rating-based study suggests that some personality structure subdomains such as “bodily self,” “affect communication,” and “use of introjects” might not be sufficiently covered by the concept of personality functioning [22••]. To our knowledge, these studies on differences of personality functioning, compared to personality organization [41••] and personality structure [22••], are the only of their kind. Yet they highlight the importance of studying their differential aspects, as they point to the possibility that these concepts each might entail unique facets that complement the respective other concepts.

## Clinical Observations

### Case Vignette

A 26-year-old man sought psychiatric consultation after his girlfriend separated from him. Since the breakup, he described depressive symptoms, lack of motivation and drive, aggressive verbal outbursts towards his family, inability to work, and an emotional state “between frustration and anger” against an unfair world in which he “has never really had a chance.” After expressing suicidal ideas and plans and an enduring feeling of being worthless, mistreated, and a refusing attitude, his family urged him to consult a clinician.

For a preliminary diagnostic approach, SCID-I [202] and SCID-II [203] were performed to assess mental disorders including categorical PDs. Besides the diagnosis of a severe major depression episode, the patient described subthreshold features of schizoid, paranoid, borderline, and obsessive-compulsive PD. Unlike the clinical impression, he denied most items for grandiose pathological narcissism but mentioned a noticeable, yet not clinically relevant, number of antisocial features. For research purposes, both STIPO and SCID-5-AMPD-I interviews were then conducted. The results based on scale scores are depicted in Table 2.

**Table 2:** Comparison of categorical and dimensional personality disorder diagnosis based on a case vignette

SCID-II		STIPO		SCID-5-AMPD-I	
Personality disorder	Criteria met	Scale	Impairment	Scale	Impairment
Antisocial^a^	2	Overall	Borderline I	Overall	Moderate
Avoidant^b^	0				
Borderline^c^	4	Identity	Moderate		
Dependent^d^	0	Object relations	Moderate	Identity	Moderate
Histrionic^d^	1	Primitive defenses	Severe	Self-direction	Mild-moderate
Narcissistic^c^	4	Coping and rigidity	Severe	Empathy	Moderate
Obsessive-compulsive^e^	3	Aggression	Moderate	Intimacy	Moderate
Paranoid^b^	3	Moral values	Mild		
Schizoid^b^	2	Reality testing	Mild		
Schizotypal^c^	0				

A minimum of ^a^3 of 7, ^b^4 of 7, ^c^5 of 9, ^d^5 of 8, or ^e^4 of 8 criteria must be met for the respective categorial disorders diagnosis. Note that none of the specific categorical personality disorder diagnoses, captured with SCID-II, were met, while the threshold for a dimensional personality disorder diagnosis was exceeded, assessed with STIPO (“Borderline Personality Organization I”) and SCID-5-AMPD-I (“moderate impairment in personality functioning”)

*SCID-II* Structured Clinical Interview for DSM-IV–Axis II disorders, *STIPO* Structured Interview of Personality Organization, *SCID-5-AMPD-I* Structured Clinical Interview for the DSM-5 Alternative Model for Personality Disorders–Module I

### STIPO Results

The patient was diagnosed at the BPO I level which represents moderate personality impairment and marks the threshold for severe PDs according to the model of personality organization. Main fields of impairment in the domain *identity* were a chronically reduced self-esteem with fluctuating self-evaluations from idealization to derogation, a sense of entitlement, a reduced ability to invest in work and leisure activities, as well as a superficial concept of other people. *Interpersonal relationships* seemed to have an exploitative background, aiming to fulfill the patient’s narcissistic needs by engaging in manipulative behaviors. He also described difficulties in experiencing sexual lust and feelings of closeness with his partner at the same time, as well as feelings of envy and a reduced sense of reciprocity. Predominantly *primitive defenses* such as splitting, projection, externalization, and the narcissistic defense of engaging in grandiose fantasies were present to regulate internal and external distress, thereby lacking adaptive coping strategies to successfully handle distressing situations. As a result, he described feeling overwhelmed easily and a tendency to stagnate while fulfilling tasks due to an overdrawn claim for perfection. Aggression was, thus, described as a predominant affect, mainly directed towards others in occasional anger outbursts with verbal force. Instead of being independently anchored in the self, the patient’s concept of *moral values* seemed rigidly based on fear of punishment and shame. Even though being overall intact, the abilities of *reality testin*g showed tendencies of paranoid expectations and envy in close relationships.

### SCID-5-AMPD-I Results

The patient displayed an overall level of personality functioning of 2 (“moderate impairment”), thus fulfilling Criterion A of the AMPD for the presence of a PD. In the section of *identity*, he showed an impaired ability to describe himself coherently and a fluctuation between grandiose and vulnerable appraisals of himself. There were indications of emotionally impulsive and erratic behaviors, oscillating between extreme negative and positive emotions, occasionally altered by a feeling of emptiness. In the section of *self-direction*, he reported to be able to set goals but to not follow them through until reaching success. He described himself to be living by prosocial standards of behavior, lacking insight in his own internal self and the ability for taking on a meta-perspective while experiencing strong emotions. In the section of *empathy*, he presented himself apprehensive and sensible towards other people’s motivations but finding himself recurrently engaged in fights. He also described a conviction of his own perspective of being superior to other people’s opinions, and a reduced ability to understand and reflect the effects of his own behavior on others. In the section of *intimacy,* he was able to describe some stable relationships; however, closer descriptions seemed to lack depth, reliance, and emotional closeness, even though he claimed to seek for this. A mutuality of regard was described, which, however, was not validated in his behaviors.

### Summary

While the patient did not endorse the necessary number of criteria to fulfill any specific DSM-IV PD, he demonstrated moderate impairment in personality functioning and a corresponding level of BPO I. Unlike his denial of most of the symptom-based, categorical questions of the SCID-II, he was more open to speak about his underlying difficulties regarding himself and his relationships with others in the STIPO and SCID-5-AMPD-I. In these interviews, he described relevant problems regarding the self (e.g., regulation of self-esteem and emotion regulation) and others (e.g., reciprocity, capacity to invest in close relationships). Using the findings from the STIPO profile, a treatment plan could be easily developed by the clinician, targeting the underlying deficits in personality organization. This case example demonstrates how specific contents of personality pathology could be picked up beyond the categorical DSM approach by both interviews, and point to the importance of unconscious dimensions of personality pathology (e.g., use of defense mechanisms) for the assessment of severity [41, 204–206].

## Conclusion

The present review focused on the dimensional concepts of personality functioning, personality structure, and personality organization in contrast to the current categorical PD classification. The three concepts are based on the principle of defining core pathological dimensions of PDs based on disturbances in the self and interpersonal relations. They differ in their theoretical foundation: while concept of personality organization is directly based in object relations theory, the concept of personality structure derives from a broader psychodynamic conceptual framework. The novel concept of personality functioning, in turn, is informed by divergent theoretical and conceptual approaches (including object relations theory) (cf. Table 1).

Based on numerous findings, it can be concluded that all three concepts have a strong empirical background, can be assessed by a variety of validated measures, and are highly relevant for clinical practice. There is evidence of a certain degree of convergence between the dimensional approaches and the categorical PD classification. While initial findings also point to advantages of the dimensional concepts over the categorical system, more studies on this subject are needed to draw definite conclusions.

While there is compelling evidence that personality functioning, personality structure, and personality organization are related to each other, it is also important to note that initial studies indicate that some differences between these concepts can be determined empirically. Clearly, the three concepts are not interchangeable. In particular, the decision as to which of the concepts to use in clinical practice will depend both on the background of the person employing them and the treatment implications of the assessment. Utilizing interviews embedded in each clinician’s individual background to assess personality pathology is helpful to develop an appropriate treatment plan [23•].

Personality structure [91] and personality organization [23•] are closely linked to psychodynamic therapeutic approaches. Consequently, clinicians with a focus on psychodynamic models may be drawn to these frameworks. One advantage of instruments assessing personality structure and personality organization is that they provide operationalizations of psychodynamic concepts that are otherwise difficult to grasp. Specifically, the operationalization of personality structure addresses a broader psychodynamic approach, whereas personality organization uses constructs directly derived from object relations theory. Depending on the respective therapeutic approach, a corresponding diagnostic instrument may be preferable. Another strength of personality structure and personality organization is the inclusion of unconscious mental processes which are crucial for psychodynamic case conceptualizations. A limitation is the necessity to be familiar with psychodynamic theory.

Personality functioning is not associated to a specific theoretical framework and is thus more accessible to other (non-psychodynamic) treatment modalities. It strengthens the approach of diagnosing personality by its functional impairments and obtaining a profile of facets of personality functioning. This background not rooted in a specific theory can be seen as a disadvantage for clinicians seeking a treatment plan related to a specific treatment model while being attractive for clinicians from various backgrounds.

As many studies have illustrated that the course of therapy is often complicated by unrecognized underlying PDs [207], and that patients with PDs have functional impairments [75••, 182, 187], it becomes clear that not only categorical PDs but also dimensional personality pathology should be carefully examined. In sum, a differentiated and dimensional diagnosis, combining a symptom-oriented approach and a dimensional approach focusing on the functional abilities and impairments of personality, can provide a broad basis for a therapeutic plan.

A clinical case vignette demonstrated the diagnostic benefit of a dimensional assessment compared to a categorical approach. Comparing the diagnostic focus derived from the three interview approaches, it became clear that relying solely on a categorical diagnosis does not take the significance of impairments in self functioning and the resulting interpersonal consequences adequately into account. These could be brought out by the STIPO and the SCID-5-AMPD-I results and be of further use for clinical case conceptualization and treatment planning.
